# Therapeutic Potential of Phenolic Compounds in Medicinal Plants—Natural Health Products for Human Health

**DOI:** 10.3390/molecules28041845

**Published:** 2023-02-15

**Authors:** Wenli Sun, Mohamad Hesam Shahrajabian

**Affiliations:** Biotechnology Research Institute, Chinese Academy of Agricultural Sciences, Beijing 100081, China

**Keywords:** phenolics, curcumin, protocatechuic, quinones, stilbenes, curcuminoids

## Abstract

Phenolic compounds and flavonoids are potential substitutes for bioactive agents in pharmaceutical and medicinal sections to promote human health and prevent and cure different diseases. The most common flavonoids found in nature are anthocyanins, flavones, flavanones, flavonols, flavanonols, isoflavones, and other sub-classes. The impacts of plant flavonoids and other phenolics on human health promoting and diseases curing and preventing are antioxidant effects, antibacterial impacts, cardioprotective effects, anticancer impacts, immune system promoting, anti-inflammatory effects, and skin protective effects from UV radiation. This work aims to provide an overview of phenolic compounds and flavonoids as potential and important sources of pharmaceutical and medical application according to recently published studies, as well as some interesting directions for future research. The keyword searches for flavonoids, phenolics, isoflavones, tannins, coumarins, lignans, quinones, xanthones, curcuminoids, stilbenes, cucurmin, phenylethanoids, and secoiridoids medicinal plant were performed by using Web of Science, Scopus, Google scholar, and PubMed. Phenolic acids contain a carboxylic acid group in addition to the basic phenolic structure and are mainly divided into hydroxybenzoic and hydroxycinnamic acids. Hydroxybenzoic acids are based on a C6-C1 skeleton and are often found bound to small organic acids, glycosyl moieties, or cell structural components. Common hydroxybenzoic acids include gallic, syringic, protocatechuic, *p*-hydroxybenzoic, vanillic, gentistic, and salicylic acids. Hydroxycinnamic acids are based on a C6-C3 skeleton and are also often bound to other molecules such as quinic acid and glucose. The main hydroxycinnamic acids are caffeic, *p*-coumaric, ferulic, and sinapic acids.

## 1. Introduction

Medicinal plants are very important worldwide, both when used alone and as a supplement to traditional medication [1,2,3,4,5]. For many years, humans have employed plants as a source of food, flavoring, and medicines [6,7,8,9,10]. Various parts of medicinal plants such as seeds, leaves, flowers, fruits, stems, and roots are rich sources of bioactive compounds [11,12,13]. Bioactive compounds should be considered as important dietary supplements [14,15,16,17,18,19]. Polyphenols are a group of secondary metabolites involved in the hydrogen peroxide scavenging in plant cells [20]. Phenolic compounds are second only to carbohydrates in abundance in higher plants, and they display a great variety of structures, varying from derivatives of simple phenols to complex polymeric materials such as lignin [21,22,23,24,25,26]. Phenolic compounds are known for their notable potential activity against various human viruses, and phenolic compounds also have immunomodulatory and anti-inflammatory activity [27]. The most abundant phenolic compounds are phenolic monoterpenes (carvacrol and thymol) and diterpenes (carnosol, carnosic acid, and methyl carnosate), hydroxybenzoic acids (*p*-hydroxybenzoic, protocatechuic, gallic, vanillic, catechol, and ellagic), phenylpropanoic acids (*p*-coumaric, caffeic, rosmarinic, chlorogenic, ferulic, cryptochlorogenic, and neochlorogenic), phenylpropenes (eugenol), coumarins (herniarin and coumarin), flavanoes (naringenin, eriocitrin, naringin, and hesperidin), flavones (apigenin, apigetrin, genkwanin, luteolin, luteolin 7-glucuronide, cynaroside, scolymoside, salvigenin, and cirsimaritin), and flavanols (catechin, astragalin, kaempferol, methyl ethers, quercetin, hyperoside, isoquercetin, miquelianin, and rutin) [28,29].

Plant phenolics are considered promising antibiofilm and antifungal agents [30,31]. Diaz et al. [32] also reported that the levels of phenolic and flavonoid compounds were correlated with the anti-inflammatory and antioxidant activities of medicinal plants. Tukun et al. [33] reported that phenolic content is significantly connected to antioxidant activity, and halophytes have high content of nutrients and phenolic metabolites. Some of the most important phenolic compounds recognized from medicinal plants are syringic acid and gallic acid from *Moringa oleifera* [34]; gallic acid, vanillic acid, 4-hydroxybenzoic acid, and syringic acid from *Peganum harmala* [35]; rosmarinic acid from *Rosmarinus officinalis* L. and *Mentha canadensis* L. [36]; vanillin from *Thymus vulgaris* [37]; caffeic acid and *p*-coumaric acid from *Ocimum basilicum* L., *Thymus vulgaris* L., *Salvia officinalis* L., and *Origanum vulgare* L. [36]; piceatannol glucoside, resveratroloside, and piceid from *Polygonum cuspidatum* [38]; trans-rhapontin, cis-rhapontin, and trans-desoxyrhaponticin from *Rheum tanguticum* Maxim. Ex Balf. [39]; herniarin from *Matricaria chamomilla* [40]; kayeassamin I, mammeasin E, and mammeasin E from *Mammea siamensis* [41]; scopoletin, fraxetin, aesculetin, fraxin, and aesculin from *Fraxinus rhynchophylla* [42]; phyllanthin, niranthin, hypophyllanthin, nirtetralin, virgastusin, heliobuphthalmin lactone, and bursehernin from *Phyllanthus amarus* [43]; schisanchinin A, schisanchinin B, schisanchinin C, and schisanchinin D from *Schisandra chinensis* [44]; 7-methyljuglone from *Drosera rotundifolia* [45], rhein, physcion, chrysophanol, emodin, and aloe-emodin from *Rheum palmatum* and *Rheum hotaoense* [46]; curcumin, demethoxycurcumin, and bis-demethoxycurcumin from *Curcuma longa* [47]; luteolin, apigenin, orientin, apigenin-O-glucuronide, and luteolin-O-glycoside from *Origanum majorana* [48]; glycitein, genistein, formononetin, daidzein, prunetin, biochanin A and daidzin, and genistin from *Medicago* spp. [49]; kaempferol 3-O-glucoside and isorhamnetin 3-O-galactoside from *Tephrosia vogelii* [50]; rutin, kaempferol 3-O-rhamnoside, and quercetin 3-O-glucoside from *M. oleifera* [34]; gallocatechin and catechin from *Mentha pulegium* [48]; taxifolin, taxifolin methyl ether, and dihydrokaempferide from *Origanum majorana* [48]; hesperidin, naringenin-O-rhamnoglucoside, and isosakuranetin-O-rutinoside from *Mentha pulegium* [48]; and punicalagin, pedunculagin I, granatin A, ellagic acid, ellagic acid pentoside, ellagic acid glucoside, and punigluconin from *Punica granatum* [51]. Phenolic phytochemicals include flavonoids, flavonols, flavanols, flavanones, flavones, phenolic acids, chalcones, isoflavones, tannins, coumarins, lignans, quinones, xanthones, curcuminoids, stilbenes, cucurmin, phenylethanoids, and several other plant compounds, owing to the hydroxyl group bonded directly to an aromatic hydrocarbon group [52]. The classes of phenolic compounds in plants are shown in Table 1.

Phenolic acids include two subgroups, i.e., hydroxybenzoic and hydroxycinnamic acids [53]. Hydroxybenzoic acids consist of gallic, *p*-hydroxybenzoic, vanillic, protocatechuic, and syringic acid, which, in common, have the C_6_-C_1_ structure [53]. Hydroxycinnamic acids, on the other hand, are aromatic compounds with a three-carbon side chain (C_6_-C_3_), with caffeic, *p*-coumaric, ferulic, and sinapic acids being the most common [52]. Gallic acid is present in cloves (*Eugenia caryophyllata* Thunb.), while protocatechuic acid can be found in coriander (*Coriandrum sativum* L.), dill (*Anethum graveolens* L.), and star anise (*Illicium verum* Hook. f.) [54]. Caffeic acid is found among others in parsley (*Petroselinum crispum* L.), ginger (*Zingiber officinale* Rosc.), and sage (*Salvia officinalis* L.), and *p*-coumaric acid is found in oregano (*Origanum vulgare* L.), basil (*Ocimum basilicum* L.), and thyme (*Thymus vulgaris* L.) [54]. Some samples of hydroxybenzoic and hydrozycinnamic acids are presented in Table 2.

Flavonoids include the largest group of plant phenolics, responsible for over half of the eight thousand naturally occurring phenolic constituents [55,56]. Flavonoids are low molecular weight compounds, including fifteen carbon atoms, arranged in a C_6_-C_3_-C_6_ configuration [53]. The genetic structure of main classes of flavonoids are shown in Table 3.

Phenolic phytochemicals play a variety of protective roles against abiotic stresses, such as UV light, or abiotic stresses, namely predator and pathogen attacks [57]. Phenolic phytochemicals are utilized by humans to treat several ailments including bacterial, protozoal, fungal, and viral infections, inflammation, diabetes, and cancer. Biosynthesis and accumulation of polyphenol and other secondary metabolites in plants is considered as an evolutionary reaction of biochemical pathways under adverse environmental influences, i.e., biotic/abiotic limitations, including increased salinity and drought stress [58,59,60]. Some of the extraction methodologies of phenolic components from medicinal and aromatic plants are maceration, digestion, infusion, decoction, Soxhlet extraction, percolation, aqueous alcoholic extraction by fermentation, counter-current extraction, ultrasound extraction, supercritical fluid extraction, and phytonics stage. The principle factors shaping the production of phenolic components are the water supplied to plants and the time of stress exposure, and, among the various quantification methods, HPLC and colorimetric tests are the most utilized to quantify the phenolic compounds analyzed [61]. Djeridane et al. [62] reported that the phenolics in medicinal plants provide substantial antioxidant activity. A positive, significant linear connection between antioxidant activity and total phenolic content revealed that phenolic components were the dominant antioxidant constituents in medicinal plants [63,64]. Various groups of tests on phenolics indicated significant mean alterations in radical scavenging activity; tannins demonstrated the strongest activity, while most quinones, isoflavones, and lignans tested revealed the weakest activity [65,66]. The most abundant flavone in *Cytisus multiflorus* is the chrysin derivative, Kaempferol-3-*O*-rutinoside is the major flavonol in *Malva sylvestris*, and Quercetin-3-*O*-rutinoside is the principle flavonol in *Sambucus nigra* [66]. *Nepeta italica* subsp. *cadmea* and *Teucrium sandrasicum* are rich in phenolics, which indicated antioxidant and cytotoxic properties [67]. Through LC-ESI-MS analysis, five phenolic acids (quinic acid, syringic acid, gallic acid, *p*-coumaric acid, and trans-ferulic acid) and five flavonoids (catechin, epicatechin, quercetrin, rutin, and naringenin) were predominant and common in some desert shrubs of Tunisian flora (*Pituranthos tortuosus*, *Ephedra alata*, *Retama raetam*, *Ziziphus lotus*, *Calligonum comosum*, and *Capparis spinosa*) [68].

The main phenolic compounds in Matico (*Piper angustifolium* R.), Guascas (*Galinsoga parviflora*), and Huacatay were chlorogenic acid and hydroxycinnamic acid derivatives [69]. High phenolic and antioxidant activity-containing medicinal plants and species such as Chanca Piedra (*Phyllanthus nirui* L.), Yerba Mate (*Ilex paraguariensis* St-Hil), Zarzaparrilla (*Smilax officinalis*), and Huacatay (*Tagetes minuta*) have the highest anti-hyperglycemia-relevant in vitro α-glucosidase inhibitory activities with no effect on α-amylase [69]. Nineteen phenolic compounds from different groups are used in wound treatment, and the compounds are tyrosol, curcumin, hydroxytyrosol, luteolin, rutin, chrysin, kaempferol, quercetin, icariin, epigallocatechin gallate, morin, silymarin, taxifolin, hesperidin, naringin, puerarin, isoliquiritin, genistein, and daidzein [70,71,72,73]. The most important identified phenolics in *Phlomis angustissima* and *Phlomis fruticosa*, medicinal plants from Turkey, by RP-HPLC-DAD were hesperidin, catechin, kaempferol, epicatechin, eupatorin, and epigallocatechin, and chlorogenic, syringic, vanillic, *p*-coumaric, ferulic, and benzoic acids [74]. Quercetin of *Cordia dichotoma* G. Forst. (Lashusa) is the most notable phytoconstituent responsible for the therapeutic efficacy [75]. Vanillic acid, nepetin, verbascoside, and hispidulin, of *Clerodendrum petasites* S. Moore (CP) were chosen as potential phenolic active compounds in Thai traditional medicine for the treatment of different kinds of skin diseases [76,77,78]. Bouyahya et al. [79] reported that compounds such as terpenoids, alkaloids, flavonoids, phenolic acids, and fatty acids of *Arbutus unedo* L., *Thymus capitatus* managed diabetes by several mechanisms such as enzymatic inhibition, interference with glucose and lipid metabolism signaling pathways, and the inhibition and the activation of gene expression involved in glucose homeostasis.

*Grewia tenax*, *Terminalia sericea*, *Albizia anthelmintica*, *Corchorus tridens*, and *Lantana camara* are frequently used to treat gastroenteritis and include higher total phenolic and flavonoid contents in Namibia [80,81,82,83,84,85]. The most important phenolics identified from pomegranate are punicalin, gallic acid, ellagic acid, pyrogallol, salycillic acid, coumaric acid, vanillic acid, sesamin, and caffeic [86], and phenolic compounds have been discovered to have inhibitory effects again α-glucosidase activities [87]. Two new phenolics, leucoxenols A and B, were obtained and identified as major secondary metabolites from the leaves of *Syzygium leucoxylon* [88]. Phenolics are main phytochemicals found in *Cyathea* species, and *Cyathea* has been considered to be a potential source of novel cancer therapeutic compounds [89]. Purified phenolic compounds from the bark of *Acacia nilotica* showed insecticidal potential against *Spodoptera litura*, and they could provide substitutes to synthetic pesticides for controlling various pests [90]. Bellumori et al. [91] reported that the roots of *Acmella oleracea* L. had about twice as many phenols as the aerial parts, and caffeic acid derivatives were the main phenolic compounds in roots and aerial parts. Kaempferol was found as the most abundant phenolic compound in basil leaf extract after using an HPLC-UC method (61.4 mg.kg^−1^) [92]. Apple fruit (*Annona squamosa* L.) has a specific spatial distribution of microbes and phenolics, its peel phenolics contain antimicrobial activity against several Gram-positive bacteria, and its peel phenolics had a growth-promoting effect toward autochthonous yeasts [93,94,95,96]. The phenolic contents of *Cyathea dregei* (root and leaves), *Felicia erigeroides* (leaves and stems), *Felicia erigeroides* (leaves and stems), *Hypoxis colchicifolia* (leaves), *Hypoxis colchicifolia* (leaves), and *Senna petersiana* (leaves) have shown high antimicrobial and cyclooxygenase (COX) inhibitory activities [97].

The most important techniques for analysis of phenolic compounds and extracts are nuclear magnetic resonance (NMR), high performance liquid chromatography (HPLC) with ultraviolet-visible (UV-Vis) or photodiode array (PDA) detector or coupled to mass spectrometry (MS), derivatization (silylation, alkylation, etc.) as well as gas chromatography (GC) or GC-MS analysis, phytochemical screening such as total flavonoid content (TFC), total phenolic content (TPC), etc., and antioxidant potential tests such as 2,2-dipehnyl-1-picrylhydrazyl (DPPH), etc. [97,98,99,100,101,102,103,104,105,106,107]. Solid-liquid extraction (SLE) is one of the main methods for extraction of phenolic compounds, specially syringic acid, catechin, and *p*-coumaric acid, which is simple, well established, and widely used [108]. Ultrasound-assisted extraction (UAE) is often used for extraction of gallic acid and rutin, which is easy to execute, uses inexpensive equipment, and consumes less solvents, and has fast extraction, good extraction yield, and low impacts on the environment [109]. Supercritical fluid extraction (SFE) usually applies for gallic acid, anthocyanin, and protocatechuic acid, which has high selectivity, cheaper and safer solvent, easily controlled extraction conditions, environmental friendliness, low operating temperature, and easy separation of solvent from solutes [110]. Microwave-assisted extraction (MAE) is used for extraction of 3-caffeoylquinic acid, 5-caffeoylquinic acid, and ellagic acid, which has short extraction time and low solvent consumption [111]. Pressurized liquid extraction (PLE) applies for extraction of rutin and quercetin, which consumes fewer organic solvents, has higher probability to avoid organic solvents by using water only, and is fast and efficient [112]. For extraction of proanthocyanidin, naringin, and hesperidin, enzyme-assisted extraction (EAE) is proposed, which is safe and green and does not need complex paraphernalia [113]. Key points about phenolic acids and their derivatives are shown in Table 4. This work aims to provide an overview of phenolic compounds and flavonoids as potential sources of pharmaceutical and medical application from recently published studies, as well as some interesting directions for future research.

## 2. The Important Health Benefits of Phenolic Components

Flavonoids and phenolics are commonly known as the largest phytochemical molecules with antioxidant characteristics [124]. Traditional Chinese medicinal plants that contain phenolic acids and flavonoids have shown high antioxidant activity. *Nepeta italica* subsp. Cadmea and *Teucrium sandrasicum* are rich in phenolic, tannin, and flavonoids content, which showed antioxidant and cytotoxic properties. *Bauhinia variegata* L. contained flavonoid compounds and revealed antioxidant properties against oxidative damage by radical neutralization, iron binding, and decreasing power abilities [125]. The rhizome extracts of *Polygonatum verticillatum* (L.) All. exhibited antioxidant activity, which is connected to the level of phenolic composition [126]. Singh and Yadav [127] have reported that, among medicinal plants, oregano, clove, thyme, and rosemary contain the highest amounts of phenolic compounds. Flavan-3-ol oligomers and monomers were potent antioxidant compounds abundantly identified in *Camellia fangchengensis* [128].

*Bellis perennis* L. was rich in phenolic compounds, and it can be used for wounds, cancer, inflammation, and eye diseases [129]. A total of 27 kinds of phenolic compounds were identified by HPLC-ESI-QTOF-MS/MS, and okra (*Abelmoschus esculentus*) polyphenols exhibited great antioxidant activity in vitro [130]. The *Althaea officinalis* extracts showed stronger antioxidant activity and excellent α-glucosidase, 5-lipoxygenase, and nitric oxide inhibitory properties [131]. *Dendrobium densiflorum* was rich in flavonoid, alkaloid, and antioxidant activity, *Acampe papillosa* was rich in total phenol, total tannin, and total saponin content, and *Coelogyne nitida* exhibited higher antioxidant activity because of its higher quercetin content [132]. Cirak et al. [133] showed that *Achillea arabica* Kotschy is an important source of natural antioxidants. The antioxidant property and bioactive constituents from the fruits of *Aesculus indica* (Wall. Ex Cambess.) Hook, which were quercetin and mandelic acid, were the major bioactive molecules with notable antioxidant properties to decrease oxidative stress caused by reactive oxygen species (ROS) [134]. The phytochemical compounds and biological activity of *Pinus cembra* L. contain higher concentration of total phenolics and flavonoids than that of needle extract, and its bark extract showed better ability as a free radical scavenger [135]. Higher antioxidant activity in normal-tannin lentil seed coats than low-tannin ones was reported; kaempferol tetraglycoside was dominant in low-tannin seed coats, and procyanidins, kaempferol tetraglycoise, and catechin-3-*O*-glucoside in normal-tannin has been found [136]. Zhang et al. [137] also reported that antioxidant activity and prebiotic impacts were positively correlated for oat phenolic compounds. 3,4-dihydroxybenzoic, rutin, vanillic acid, and quercetin were detected from aqueous extracts of *azendjar* and *taamriouth* figs, and a dark peel variety consisted of more phenolics and exerted a higher antioxidant capacity [138]. Although gallic acid was the most important compound in carob (*Ceratonia siliqua* L.) pulp extract, geographic origin strongly influenced the contents of bioactive compounds and antioxidant activities [139].

*Asplenium nidus* L. contained gliricidin 7-*O*-hexoside and quercetin-7-*O*-rutinoside that can fight against three pathogens, i.e., *Proteus vulgaris* Hauser, *Proteus mirabilis* Hauser, and *Pseudomonas aeruginosa* (Schroeter) Migula [140]. Flavones, which were extracted from the root of *Scutellaria baicalensis* Georgi, were proven as potential antibacterial agents against *Propionibacterium acnes*-induced skin inflammation both in in vitro and in vivo models [141]. Kaempferol that was isolated from the *Impatiens balsamina* L. exhibited potential activity to inhibit the growth of *P. acnes* [142]. Phenolics from kernel extract *Mangifera indica* L. also showed anti-acne properties to inhibit the growth of *P. acnes* [143]. Medicinal plants such as *Albizia procera*, *Atalantia monophylla*, *Asclepias curassavica*, *Azima tetracantha*, *Cassia fistula*, *Costus speciosus*, *Cinnamomum verum*, *Nymphaea stellata*, *Osbeckia chinensis*, *Punica granatum*, *Piper argyrophyllum*, *Tinospora cordifolia*, and *Toddalia asiatica* have shown antifungal activity [144]. The strictinin isolated from the leaves of *Camellia sinensis* var. *assamica* (J.W. Mast.) Kitam was a good substitute for antibacterial activities [145]. Phenolic compounds, especially flavonoids, have long been reported as chemopreventive factors in cancer therapy [146,147,148]. The extract of *Curcuma longa* L. rhizome has been suggested as a promising source of natural active compounds to fight against malignant melanoma due to its potential anticancer property in the B164A5 murine melanoma cell line [149]. Glircidia 7-*O*-hexoside and Quercetin 7-*O*-rutinoside, which were flavonoids isolated from the medicine fern (*Asplenium nidus*), were also proposed as potential chemopreventives against human hepatoma HepG2 and human carcinoma HeLa cells [140]. Quercetin can induce miR-200b-3p to regulate the mode of self-renewing divisions of the tested pancreatic cancer [150], and a soy isoflavone genistein inhibited the activation of the nuclear factor kappa B (NF-KB) signaling pathway that maintains the balance of cell survival and apoptosis; this soy isoflavone could also take its action to fight against cell growth, apoptosis, and metastasis, including epigenetic modifications in prostate cancer [151]. Curcumin exhibits anticancer impacts towards skin cancers, as this phenolic can influence the cell cycle by acting as a pro-apoptotic agent [152]. Curcumin acts as a non-selective cyclic nucleotide phosphodiesterase (PDE) inhibitor to inhibit melanoma cell proliferation, which is associated with epigenetic integrator UHRF1 [153]. Curcumin inhibited proliferation of the selected cell lines in prostate cancer and induced apoptosis of the cancer cells with a dose-dependent response [154].

The cardioprotective impacts from various kinds of phenolics and flavonoids occurring in medicinal plants have been investigated in many studies [155,156]. Many phenolic and flavonoid compounds have been studied and had reported their cardioprotective properties via different mechanisms including inhibition of ROS generation, apoptosis, mitochondrial dysfunction, NF-KB, p53, and DNA damage both in vitro and in vivo, and clinical studies [157]. Kaempferol, luteolin, rutin, and resveratrol showed their efficacy against doxorubicin-induced cardiotoxicity [158,159]. Isorhamnetin provided a cardioprotective effect against cardiotoxicity of doxorubicin and potentiated the anticancer efficacy of this drug [160]. The total phenolic and flavonoid contents of the aqueous fraction from *Marrubium vulgare* L. have effects on ischemia-reperfusion injury of rat hearts, which proved that the aqueous fraction from *M. vulgare* had cardioprotective potential [156]. Aspalathin and phenylpyruvic acid-2-*O*-β-D-glucoside, two of the major compounds from *Aspalathus linearis* (Burm.f.) R. Dahlgren, were demonstrated as potential protective compounds to protect myocardial infarction caused by chronic hyperglycemia [155]. Puerarin is a potential isoflavone that was reported as an interesting candidate for cardioprotection by protecting myocardium from ischemia and reperfusion damage by means of opening the Ca^2+^-activated K^+^ channel and activating the protein kinase C [161]. Quercetin, hesperidin, apigenin, and luteolin were reported as flavonoids containing potential anti-inflammatory impacts [162]. The flavonoids and phenolic compounds of *Phyllanthus acidus* leaves could be correlated with the analgesic, antioxidant, and anti-inflammatory activities [163]. Hydroxytyrosol and quercetin 7-*O*-α-L-rhamnopyranoside exhibited anti-inflammatory activity through lowering the levels of TNF-α, and hydroxytyrosol and caffeic acid showed significant anti-inflammatory activity at 100 μm by reducing the release of NO in LPS-stimulated macrophages comparable to positive control indomethacin [164].

The most important chemical compounds extracted from ethanol of *Cardiospermum halicacabum* were chrysoeriol, kaempferol, apigenin, luteolin, methyl 3,4-dihydroxybenzoate, 4-hydroxybenzoic acid, quercetin, hydroquinone, protocatechuic acid, gallic acid, and indole 3-carboxylic acid, which have shown high anti-inflammatory and antioxidant activities [165]. The most important phenolic components with antiviral effects against COVID-19 were curcumin, Theaflavin-3,3′-digallate, EGCG, Paryriflavonol A, Resveratrol, Quercetin, Luteolin, Scutellarein, Myricetin, and Forsythoside A [166]. In traditional Persian medicinal science, medicinal plants such as *Glycyrrhiza glabra* L., *Rheum palmatum* L., *Punica granatum* L., and *Nigella sativa* L. have been introduced for treating respiratory disorders and infections because of their phenolic compounds [167]. The anti-inflammatory activity of polyphenolic compounds in *Gaillardia grandiflora* Hort. Ex Van Houte and *Gaillardia pulchella* Foug from Egypt were reported [168]. Anti-inflammatory properties of two medicinal plant species, *Bidens engleri* O.E. Schulz from Asteraceae family as well as *Boerhavia erecta* L. from Nyctaginaceae family, were identified and reported in various fractions [169]. *Plantago subulata* has shown anti-inflammatory properties on macrophages and a protective effect against H_2_O_2_ injury [170]. Phenolic content changes with aromatic and medicinal plant species and extraction method used [171]. Astilbin, a dihydroflavonol, from *Smilax glabra* Roxb significantly inhibited nitric oxide production, tumor necrosis factor-α (TNF-α), and mRNA expression of inducible nitric oxide synthase in the tested cells [172]. Apigenin is a main flavone with skin protective impact against UV light; this flavone can be identified in various edible medicinal plants or plants-derived beverages, e.g., beer, red wine, and chamomile tea [173,174]. Quercetin is a flavonol that can be discovered in apple peel, onion skin, and *Hypericum perforatum* L. leaves [175]. Silymarin, a standardized extract of flavonolignans from the milk thistle (*Silybum marianum* (L.) Gaernt.) fruits, consists of silybin, a principle active component [176]. Genistein is a soybean isoflavone that was also reported as photoprotective molecule against photocarcinogenesis by inhibiting UV-induced DNA damage in human skin-equivalent in vitro model [177]. Equol is considered as an isoflavonoid metabolite from isoflavone daidzein or genistein produced by gut microflora [178,179]. Genistein is an obvious example of an interesting choice of a flavonoid phytoestrogen for improving endothelial roles in postmenopausal women with MetS [180]. A chrysin derivative was the most abundant flavone in *Cytisus multiflorus*, quercetin-3-*O*-rutinoside was the main flavonol in *Sambucus nigra,* and kaempferol-3-*O*-rutinoside was the main flavonol in *Malva sylvestris* [181]. Biological properties of phenolic compounds are presented in Table 5.

## 3. Hydroxybenzoic Acids (Gallic Acid and Protocatechuic Acid)

Hydroxybenzoic acids (HBAs) are antioxidant phytochemicals found in many medicinal plants and are efficient for prevention of various human diseases [247,248]. Joshi et al. [249] reported that 4-hydroxybenzoic acid (4HBA) is a potential antidiabetic, anticancer, antifungal, antioxidant, and cardioprotective, etc. *Piper garagaranum* C. DC contains prenylated hydroxybenzoic acids, and prenylated hydroxybenzoic acids indicated anti-inflammatory characteristics, as determined in murine macrophage assays [250].

### 3.1. Gallic Acid

Gallic acid is one of the most abundant polyphenols identified in nature [251,252]. Behera et al. [253] reported that gallic acid reveals antioxidant or free radical scavengers in adipocyte proliferation. Gallic acid is found in a wide range of natural plants, it is associated with the health of human beings, and it has well-documented anticancer, antibacterial, anti-inflammatory, and antifungal activities [254,255]. Gallic acid in *Emblica officinalis* mediated antidiabetic potential and delineated the upregulation of pAkt, PPAR-γ, and Glut4 through gallic acid-mediated antidiabetic properties, thus providing potent therapy for diabetes [256]. Gallic acid inhibited about 44–57% of the total CaOx crystal formations, and it is a promising agent with antiurolithiatic properties for the treatment and prevention of urinary or kidney stones [257]. Gallic acid supplementation adjusted serum lipid metabolism by decreasing serum triglyceride, fat digestibility, and bacteroidetes/firmicutes ratio [258]. Gallic acid prevents the development and occurrence of gastric precancerous lesions (GPL) by inhibiting the Wnt/β-catenin signaling pathway and then suppressing the epithelial–mesenchymal transition (EMT) process [259]. Gallic acid is a direct thrombin inhibitor with a platelet aggregation inhibitory effect [260]. Gallic acid shows significant binding and disruption of protease structure, and gallic acid has a potential phytotherapeutic effect against fungal protease, which is a notable virulence factor [261]. Gallic acid can boost gut microbiota alterations connected with cardiovascular disease (CVD) and suggests that males suffering from atherosclerosis may benefit from gallic acid supplementation, as this polyphenol partially restored microbiome dysbiosis [262]. Gallic acid could decrease the noxious impacts of diclofenac (DIC) on the antioxidant defense system and renal tissue [263].

### 3.2. Protocatechuic Acid

Protocatechuic acid (3,4-dihydroxybenzoic acid) is a natural phenolic acid, and one of the chief metabolites of complex polyphenols [264]. It can be identified in many plants such as bran and grain brown rice, particularly in the scales of onion, plums, grapes, gooseberries, and nuts such as ordinary almonds [265,266]. Da-Costa-Roch et al. [267] and Adedara et al. [268] reported that protocatechuic acid can be found in many medicinal plants, especially *Hibiscus sabdariffa* L. (Hs, roselle; Malvaceae). Protocatechuic acid has different activities such as neuroprotective activities, antiosteoporotic activities, antitumor activities, and the protective effects against hepatotoxic and nephrotoxic activities [269,270]. It has also antibacterial, antiulcer, anti-aging, antidiabetic, anticancer, antiviral, antifibrotic, analgesic, anti-inflammatory, anti-atherosclerotic, and cardiac activity [271,272]. Protocatechuic acid from bitter melon (*Momordica charantia*) alleviates cisplatin-induced oxidative renal damage, which proves it has protective activity against anticancer drug-induced oxidative nephrotoxicity [273]. Protocatechuis acid inhibits Cd-induced neurotoxicity in rats, increases the Nrf2 signaling pathway, and exhibits anti-apoptotic and anti-inflammatory activities [274]. *Veronica montana* has protocatechuic acid as the main phenolic molecule, and it kills bacteria by affecting its cytoplasmic membrane [275].

## 4. Hydroxycinnamic Acids (*p*-Coumaric Acid, Caffeic Acid, Ferulic Acid, Sinapic Acid)

Hydroxycinnamic acid derivatives are a notable class of polyphenols found in vegetables, fruits, and medicinal plants, and extensively consumed in human diet [276,277]. Hydroxycinnamic acids significantly contribute to antioxidant capacity [278]. Hydroxycinnamic acids are widely found in plants and their products such as cereals, fruits, coffee, vegetables, etc. [279,280].

### 4.1. p-Coumaric Acid

*p*-Coumaric acid is a plant metabolite with antioxidant and anti-inflammatory impacts [281,282]. *p*-Coumaric acid boosts hepatic fatty acid oxidation and fecal lipid excretion, and it affects inflammatory and insulin resistance-related adipokines. *p*-Coumaric acid stimulates electrical factors of biological and model lipid membranes [283].

### 4.2. Caffeic Acid

Caffeic acid (3,4-dihydroxycinnamic acid) has been known as an important source of natural antioxidants in different agricultural products [284,285]. It has immense use in cancer treatment [286,287], and it could be known as an important natural antioxidant [288]. Caffeic acid can induce apoptosis in cancer cells through increasing ROS levels and impairing mitochondrial function, and it also benefits from reducing aggressive behavior of tumors via suppressing metastasis [289]. Caffeic acid has anti-inflammatory and antioxidant properties against 6-propyl-thiouracil (PTU)-induced hypothyroidism [290]. Meinhart et al. [291] reported that higher sums of mono-caffeoylquinic acids were found in mulberry, quince, and bilberry, and the dicaffeoylquinic acids sum was higher in granadilla, passion fruit, and kumquat. It is a phenolic compound extensively discovered in commonly consumed foods such as apples, pears, and coffee [292]. The biosynthesis pathway of caffeic acid can be categorized into two modules, (1) L-tyrosine is synthesized from carbon sources via the glycolytic pathway, the pentose phosphate pathway, and the shikimate pathway; (2) caffeic acid is generated by the continuous deamination and hydroxylation of L-tyrosine [293]. Trifan et al. [294] found that caffeic acid oligomers reported in *Symphytum officinale* L. root may contribute to the anti-inflammatory activity for which comfrey preparations are used in traditional medicine. Caffeic acid phenethyl ester extracted from *Rhodiola sacra* could provide health benefits, decreasing the magnitude of the inflammatory process triggered by endotoxin shock and the production of inflammatory mediators [295]. Caffeic acid from the leaves of *Annona coriacea* have shown antidepressant-like impacts, which involve important neurotransmitter systems [296]. Spagnol et al. [297] reported that caffeic acid presented antioxidant activity greater than ascorbic acid and trolox. Caffeic acid regulates lipogenesis-related protein expression in high-fat diet (HFD)-fed mice, alleviates endotoxemia and the proinflammatory response in HFD-fed mice, and attenuates gut microbiota dysbiosis in HFD-fed mice [298]. Caffeic acid decreases oxidative stress levels in the hippocampus and regulates microglial activation in the hippocampus [299].

### 4.3. Ferulic Acid

Ferulic acid (4-hydroxy-3-methoxycinnamic acid) is a polyphenol that is widely known for its therapeutic potential, showing anti-aging, anti-inflammatory, and neuroprotective impacts [300,301]. The ferulic acid molecule reveals cis-trans isomerism, with the most abundant form in nature being the trans isomer, and both isomers have proven results in the treatment of several pathologies such as diabetes, cancer, and neurodegenerative and cardiac diseases [302]. Ferulic acid is important for the synthesis of significant chemical molecules such as coniferyl alcohol, di ferulic acid, vanillin, synaptic, and curcumin, as well as for giving the cell wall stiffness [303]. Ferulic acid can be applied as an antioxidant to prevent damage from ultraviolet (UV) radiation and skin carcinogenesis [304]. It is ample in numerous fruits and vegetables, including bananas, eggplant, citrus fruits, and cabbage, as well as in seeds and leaves [305,306]. In Chinese medicinal science, ferulic is normally joined with polysaccharides by covalent bonds in various plant cell walls such as cereal bran and regarded as the main bioactive compound of *Angelica sinensis*, chuanxiong rhizoma, and ferula [307], and it has several biological activities such as anti-apoptosis, anticancer, antioxidant, and anti-inflammatory impacts [308]. Free ferulic acid is related to the natural content of ferulic acid in herbs, and total ferulic acid refers to the sum of free ferulic acid plus the amount of related hydrolyzed components [309,310]. *Angelica sinensis* is a perennial herbaceous species that creates the bioactive metabolite ferulic acid [311,312]. The ferulic compounds of *Salvia officinalis* could be useful as a safe natural source for estrogenic characteristics [313]. Singh et al. [314] indicated that ferulic acid is a phenol derivative from natural sources and applied it as a potential pharmacophore that exerts multiple pharmacological properties such as neuroprotection, Aβ aggregation modulation, antioxidant, and anti-inflammatory. Ferulic acid increases cerebellar functional and histopathological changes induced by diabetes, which can be attributed to its antioxidative effect and its ability to modulate nitric oxide synthase (NOS) isoforms [315]. Ramar et al. [316] showed that ferulic acid and resveratrol revealed antioxidant as well as antidiabetic effects, consequently modulating liver, kidney, and pancreas damage caused by alloxan-induced diabetes, possibly via inhibition of the proinflammatory factor, NF-KB. Ferulic acid treatment prevents radiation-induced lipid peroxidation and DNA damage and restores antioxidant status and histopathological alterations in experimental animals [317]. Hu et al. [318] found that ferulic acid could alleviate inflammation and oxidative stress. Ferulic acid can inhibit cancer proliferation through various mechanisms, including changing the cancer cell cycle, inducing apoptosis, and regulating proteins involved in cell proliferation [319], and ferulic acid could be used as a potential official adjuvant for breast cancer treatment [320].

### 4.4. Sinapic Acid

Sinapic acid, a widely prevalent hydroxycinnamic acid, contains numerous biological activities related to its antioxidant property [321,322]. It protects lysosomes and prevents lysosomal dysfunction [323]. Saeedavi et al. [324] reported that sinapic acid may be a new therapeutic potential to treat allergic asthma through suppressing T-helper 2 immune responses. Sinapic acid phenethyl ester boosts gene expression related to the cholesterol metabolic process [325]. Hu et al. [326] indicated that sinapic acid can be utilized as an effective chemo preventive agent against lung carcinogenesis. It can also alleviate blood glucose levels by improving insulin production in pancreatic β-cells, and it can exhibit an antioxidative impact by suppressing lipid peroxidation and increasing the activity of antioxidant enzymes [327]. Sinapic acid significantly increases caspase-3 activity and inhibits cell invasion, and it has anticancer impacts on prostate cancer cells [328]. Sinapic acid pretreatment mitigates renal impairment and structural injuries through the downregulation of oxidative/nitrosative stress, inflammation, and apoptosis in the kidney [329]. Raish et al. [330] indicated the ability of sinapic acid to restore the antioxidant system and to suppress oxidative stress, pro-inflammatory cytokines, extracellular matrix, and TGF-β, and showed that sinapic acid treatment (10 and 20 mg/kg) significantly ameliorated bleomycin (BML)-induced lung injuries. Singh and Verman [331] revealed that sinapic acid increases streptozotocin (STZ)-induced cognitive impairment by ameliorating oxidative stress and neuro inflammation in the cortex and hippocampus. Sinapic acid can modulate the redox state in high-fat diet (HFD) rats [332].

## 5. The Health Benefits of Coumarins (Umbelliferone, Esculetin, Scopoletin)

Coumarins (2*H*-chromen-2-one ring) with the molecular formula C_9_H_6_O_2_ are an important group of natural compounds and are used as additives in both cosmetics and foods [333], and they constituent a notable class of heterocyclic compounds with the characteristic benzo-α-pyrone moiety in its structure [334]. Coumarin has been reported to have antibacterial, anticancer, antioxidant, anti-inflammatory, anticoagulant, and anti-Alzheimer’s disease (AD) activities [335,336]. Coumarin derivatives are found naturally as secondary metabolites in more than 150 species of plants and in over 30 plant families such as *Clusiaceae*, *Umbelliferae*, *Guttiferae*, *Rutaceae*, *Oleaceae*, *Fabaceae*, and many more [337]. Seo et al. [338] reported that different coumarins were identified from the roots of *Angelica dahurica* using NMR spectroscopy, and each coumarin revealed remarkable differences in content and inhibitory effect. Kassim et al. [339] indicated that the good antioxidant activity of *Melicope glabra* (Rutaceae) is because of umbelliferone, glabranin, and scopoletin. Coumarin-based compounds extracted from the medicinal plants are shown in Table 6.

### 5.1. Umbelliferone

Umbelliferone is a 7-hydroxycoumarin and an isomer of caffeic acid [341], and it has been reported for different pharmacological activities against numerous diseases such as cancer [342]. The plant sources of umbelliferone are *Acacia nilotica*, *Angelica decursiva*, *Aegle marmelos*, *Artemesia tridentata*, *Aster praelatus*, *Balsamocitrus camerunensis*, *Chamomilla recutita*, *Citrus aurantium*, *Cirtus natsudaidai*, *Citrus paradise*, *Coriandrum sativum*, *Diospyros oocarpa*, *Diplostephium foliosissimum*, *Dystaenia takeshimana*, *Edgeworthia chrysantha*, *Edgeworthia gardneri*, *Eriostemon apiculatus*, *Ferula communis*, *Ferula communis*, *Ferula assafoetida*, *Fructus Aurantii*, *Glycyrrhiza glabra*, *Angelica archangelica*, *Haplophyllum villosum*, *Harbouria trachypleura*, *Haplopappus desertzcola*, *Haplophyllum patavinum*, *Hydrangea chinensis*, *Hydrangea macrophylla*, *Hieracium pilosella*, *Ipomoea mauritiana*, *Justicia pectoralis*, *Matricaria recutita*, *Melicope glabra*, *Musa* spp., *Parkinsonia aculeata*, *Peucedanum praeruptorum*, *Picea abies*, *Potentilla evestita*, *Rhododendron lepidotum*, *Platanus acerifolia*, *Selaginella stautoniana*, *Saussurea eopygmaea*, *Stellera chamaejasme*, and *Typha domingensis* [343]. It has been reported to have antioxidant, anti-inflammatory, free radical scavenging, and antihyperglycemic properties [344], and antifungal characteristics [345]. Althunibat et al. [346] reported that umbelliferone prevented isoproterenol cardiotoxicity in rats, and it decreased isoproterenol-induced oxidative stress and inflammation. Kutlu et al. [347] reported that umbelliferone has a strong antioxidant and anti-inflammatory effect on sepsis, and it can be considered as a new treatment for organ dysfunction. Umbelliferone ameliorates atopic dermatitis (AD)-associated symptoms and inflammation via regulation of various signaling pathways, suggesting that umbelliferone might be a potential therapeutic of AD [348]. Umbelliferone downregulates TGF-β1 levels in kidney tissue and it may promote kidney function and ameliorate renal oxidative stress [349]. Mohamed et al. [350] indicated that umbelliferone ameliorated oxidative stress-related hepatotoxicity via its ability to augment cellular antioxidant defenses by activating Nrf2-mediated HO-1 expression. Umbelliferone exhibits anticancer impacts on human oral carcinoma (KB) cell lines, with the increased generation of intracellular reactive oxygen species (ROS) triggering oxidative stress-mediated depolarization of mitochondria [351]. Umbelliferone has gastric protective activity in vivo, and it has antidiarrheal activity in vivo [352].

### 5.2. Esculetin

Esculetin (6,7-dihydroxycoumarin), a natural coumarin derived from herbs, has shown different pharmacological activities [353]. Kadakol et al. [354] reported that esculetin, a naturally occurring 6,7-dihydroxy derivative of coumarin, has revealed its potential function in various non-communicable diseases (NCDs) including obesity, diabetes, renal failure, cardiovascular disease, cancer, and neurological disorders. Esculetin reduced both chronic and acute topic skin inflammation, and mitigated inflammation by suppressing infiltration of inflammatory cells [355]. It can be found in many medicinal plants such as *Artemisia capillaris*, *Matricaria chamomilla* L., *Artemisia scoparia*, *Citrus limonia*, *Cortex Fraxini,* and *Ceratostigma willmottianum* [356,357,358]. Esculetin supplementation could protect against development of non-alcoholic fatty liver in diabetes via regulation of glucose, lipids, and inflammation [359]. The esculetin protects human hepatoma HepG2 cells from hydrogen peroxide-induced oxidative injury, and the production is provided via the induction of protective enzymes as part of an adaptive response mediated by Nrf2 nuclear accumulation [360]. Esculetin prevents progressive renal fibrosis under insulin resistance (IR) and type 2 diabetic nephropathy (T2D) conditions, and it decreases oxidative stress in the kidney under IR and T2D conditions [361]. Esculetin has the ability to suppress tumor growth and metastasis via Axin2 suppression, which can be an attractive therapeutic strategy for the treatment of metastatic colorectal cancer (CRC) [362]. Esculetin treatment decreased neurological defects and improved cognitive impairments in transient bilateral common carotid artery occlusion (tBCCAO)-treated mice, and the mechanism underlying the pharmacological impacts of esculetin involved its action on mitochondrial autophagy and the apoptosis triggered by mitochondrial oxidative stress via mediation of mitochondrial fragmentation during transient cerebral ischaemia and reperfusion injury [363]. Zhang et al. [364] reported that esculetin could be a potential therapeutic drug for the treatment of hepatic fibrosis by inducing stellate cell senescence. Wang et al. [365] indicated that esculetin is safe and reliable, is easy to be absorbed by the body, and can be synthesized in a variety of ways. Esculetin inhibits the pyroptosis of microvascular endothelial cells through the NF-KB/NLFP3 signaling pathway and is expected to be conducive in treating pyroptosis-related diseases [366]. Esculetin directly binds to hnRNPA1 and decreases the concentration of hnRNPA1 in endometrial cancer cells, and it downregulates the levels of BCL-XL and XIAP expression, resulting in apoptosis and an arrest in proliferation [367]. Esculetin inhibits clear cell renal cell carcinoma growth in a dose- and time-dependent manner, and it induces apoptosis and cell cycle arrest [368]. Esculetin could be used as a dietary therapy for the prevention of alcoholic liver disease, and it can markedly prevent ethanol-induced liver injury in mice [369].

### 5.3. Scopoletin

Scopoletin (6-methoxyl-7-hydroxy coumarin) has a phenolic hydroxyl structure and is a member of the coumarin family [370]. It has a long history of use for its medicinal characteristics in traditional Chinese medicine [371]. Scopoletin is one of the main bioactive components of *Convolvulus prostratus* Forssk, known to have a role in acetylcholinesterase inhibitor, antimicrobial, memory enhancer, and antioxidative properties [372]. It is a major component of noni (*Morinda citrifolia* L.), which contributes to the anti-inflammatory, antioxidative, immunomodulatory, and hepatoprotective properties [373]. Scopoletin could be a potential phagocytic enhancer, and it can increase immunity through enhancing macrophage phagocytic capabilities [374]. Scopoletin improved vancomycin-induced renal injury via restoring the antioxidant defense system [375]. Scopoletin reduces non-alcoholic fatty liver disease in high-fat diet-fed mice [376]. It has been reported that scopoletin could exert a positive impact on anti-aging related to autophagy via modulation of p53 in human lung fibroblasts [377].

## 6. The Health Benefits of Stilbenes (Resveratrol, Piceatannol, Pterostilbene)

Stilbenes (based on the 1,2-diphenylethylene skeleton) are a group of plant polyphenols with rich structural and bioactive diversity [378]. They originate from plant families such as Vitaceae, Gnetaceae, Leguminaceae, and Dipterocarpaceae, and, structurally, they have a C6-C2-C6 skeleton, normally with two isomeric forms [379,380]. They have wonderful potential for anti-inflammatory, antiviral, anticancer, and antioxidant activities, as well as an application as cosmetic materials, coloring agents, and dietary supplements [381,382,383]. Wine and grapes are the main dietary source of stilbenes [384]. These compounds are synthetized by plants in response to abiotic or biotic stress situations [385]. Most stilbene compounds reveal antimicrobial properties, acting as phytoalexins in response to pathogen or herbivore attack [386]. Phytochemical phenols of stilbene families indicated good stability at elevated temperatures [387].

### 6.1. Resveratrol

Resveratrol (3,5,4′-trihydroxy-trans-stilbene) is a plant polyphenol, extensively popularized during the last decades, owing to its promising beneficial effects on human health [388]. It is a famous non-flavonoid polyphenol, related to the family of stilbenes whose structure consists of two phenolic rings linked by a double bond, which promotes two isomeric conformations: trans- and cis-resveratrol [389,390]. Resveratrol’s cis-isomer is unstable, and its trans-isomer contains greater stability, but converts to the cis-isomer under exposure to high pH or UV light [391,392], with heat increasing the degradation process [391]. It exists in many traditional herbs, and in several types of fruits, especially in the muscadine grape, red wine, cranberry, lingonberry, and redcurrant [393], and roots of various plant species including *Polygonum cuspidatum* and rhubarb (*Rheun rhapontiicum*) [394]. It is also useful in common age-related diseases such as cancer, cardiovascular diseases, type 2 diabetes, and neurological conditions, and it has also positive impacts on metabolism and can boost the lifespan of various organisms [395]. Resveratrol supplementation can be considered as an adjuvant therapy for relieving inflammation [396]. It has great potency in treating cardiovascular diseases [397]. Resveratrol attenuates kidney damage in malignant hypertension rats, and it can increase glomerular filtration while decreases proteinuria [398]. It inhibits the release of proinflammatory cytokines and leads to the release of anti-inflammatory cytokines, and it scavenges free radicals and upregulates antioxidant enzymes [399]. Chowdhury et al. [400] indicated that resveratrol treatment indicated beneficial impacts on preventing oxidative stress and fibrosis in the kidneys of high-fat (HF) diet-fed rats, probably by modulating the gene expression of oxidative stress and inflammation-related parameters and enzymes. Resveratrol can downregulate the pro-inflammatory cytokine release decreasing lung injury [401]. Resveratrol-containing fruits could be a promising substitute for the management of Alzheimer’s disease [402]. It can be more effective in cardiotoxicity prevention [403]. *Polygonum cuspidatum* is an important medicinal plant in China and a rich source of resveratrol compounds, which is a secondary metabolite formed in the long-term evolution procedure of plants to increase their response to adverse environments such as pathogens and ultraviolet radiation [404]. As an anticancer parameter, resveratrol promotes apoptosis in hepatocellular carcinoma cells [405]. Bhaskara et al. [406] reported that resveratrol is a potential reducing factor that can prevent carcinogenesis due to its antioxidant abilities, and it acts as an immunomodulatory agent for treating cancer. Resveratrol can exhibit anti-aging activity through a variety of signaling pathways [407]. Resveratrol shows potent anti-rotavirus efficacy in vitro and in vivo, and it blocks viral structural expression and genomic RNA synthesis [408]. Resveratrol oligomers from *Paeonia suffruticosa* indicate neuroprotective effects in vitro and in vivo by regulating cholinergic, antioxidant, and anti-inflammatory pathways, and they may have promising applications in the treatment of Alzheimer’s disease [409]. Resveratrol is also involved in neurodegenerative diseases (NDs) with multiple neuroprotective activities [410]. Antimicrobial activity of resveratrol against many bacteria and fungi has been reported, such as antimicrobial activity against Gram-positive bacteria such as *Bacillus cereus*, *Bacillus megaterium*, *Staphylococcus aureus*, *Enterococcus faecalis*, *Enterococcus faecium*, *Mycobacterium tuberculosis*, *Mycobacterium smegmatis*, *Streptococcus pneumoniae*, *Streptococcus pyogenes*, *Propionibacterium acnes*, and *Listeria monocytogenes*; against Gram-negative bacteria such as *Escherichia coli*, *Klebsiella pneumoniae*, *Salmonella enterica serovar Typhimurium*, *Pseudomonas aeruginosa*, *Helicobacter pylori*, *Arcobacter butzleri*, *Arocobacter cryaerophilus*, *Haemophilus ducreyi*, *Neisseria gonorrhoeae*, *Neisseria meningitidis*, *Vibrio cholerae*, *Fuscobacterium nucleatum*, *Campylobacter jejuni*, and *Campylobacter coli*; and against fungi such as *Trichophyton mentagrophytes*, *Trichophyton tonsurans*, *Trichophyton rubrum*, *Epidermophyton floccosum*, *Microsporum gypseum*, *Candida albicans*, *Saccharomyces cerevisiae*, *Botrytis cinerea*, and *Trichosporon beigelii* [411]. Resveratrol has powerful anticancer characteristics in different cancer cells and organs such as pancreatic cancer, colorectal cancer, gastric cancer, esophageal cancer, hepatocellular cancer, oral cancer, and biliary tract cancer [412]. Resveratrol decreases damage to pancreatic tissue via suppression of calcium overload; it suppresses calcium overload and, thereby, decreases trypsinogen activation, oxidative stress, mitochondrial dysfunction, and disorders, and it also reduces damage to other organs such as lung and heart by decreasing microcirculatory dysfunction [413].

### 6.2. Piceatannol

Piceatannol (3,4,3′,5′-tetrahydroxy-trans-stilbene), a natural polyphenolic stilbene, has pleiotropic pharmacological potentials [414]. It can be found in different kinds of fruits and vegetables such as blueberries, grapes, and passion fruit [415]. Piceatannol is a metabolite of resveratrol found in red wine, which prevents cardiac hypertrophy in rat neonatal cardiomyocytes [416]. It has previously been known as an antileukemic principle, which has been shown to be an inhibitor of protein-tyrosine kinase activity [417]. It has been reported that its low water-solubility and bioavailability could limit its application in both food and pharmaceutical fields [418]. Piceatannol, compared with the renowned resveratrol, is a better anticancer factor and a superior agent with other biological properties [419]. Piceatannol lightened oxidative injury and collagen synthesis in lung tissues during pulmonary fibrosis, and it suppressed the activation and collagen synthesis of TGF-β-induced lung fibroblasts [420]. It appears to be an appropriate nutritional or pharmacological biomolecule that modulates effector T cell functions, namely cytokine production, differentiation, and proliferation [421]. Piceatannol attenuates fat accumulation in steatosis-induced HepF2 cells, it suppressed lipogenesis and fatty acid uptake in steatosis-induced HepG2 hepatocytes, and it suppressed fatty acid-induced oxidative stress [422]. It shows anti-aggregation activity, and it increases catalase and glutathione peroxidase activity [423]. It can also be considered as a potential chemotherapeutic factor in the treatment of leukemia, but it may be connected with the risk of multi-drug resistance [424]. Passion fruit seed extract and piceatannol could exert anticancer activity via human glyoxalase I (GLO I) inhibition [425]. Piceatannol is a promising medication for preventing acute liver failure and the mechanisms may be associated to its inhibitory impacts on ER stress, inflammation, and oxidative stress [426]. Piceatannol has a potential inhibitory activity against human glyoxapase I (GLO I), and it inhibits the proliferation of GLO I-dependent human lung cancer [427]. It protects ARPE-19 cells against apoptosis induced by photo-oxidation, and the protective effect of piceatannol is because of the activation of the Nrf2/NQO1 pathway [428]. Piceatannol is a potent enhancer of cisplatin-induced apoptosis, and it reveals the potential for clinical development for the treatment of ovarian cancer [429]. It has been reported that piceatannol significantly decreases the degree of bovine serum albumin (BSA) glycosylation, and this suggests its potential impact on preventing the progression of diabetes mellitus [430].

### 6.3. Pterostilbene

Pterostilbene, a dimethyl ester derivative of resveratrol, may act as a cytotoxic and anticancer factor [431]. It primarily exists in blueberries, grapevines, and heartwood of red sandalwood [432,433]. Phenolic resveratrol, pterostilbene has been reported to have antifungal activity against a broad range of important phytopathogenic fungi such as *Leptosphaeria maculans* and *Peronophythora litchii* [434]. It is an anti-inflammatory and antioxidant agent with preventive effects toward skin disorders, and its anticancer impacts include inducing necrosis, apoptosis, and autophagy [435]. It can alleviate hepatic damage and oxidative stress and increase hepatic antioxidant function in piglets [436]. It possesses the abilities of antiproliferation, reversing epithelial to mesenchymal transition (EMT), and suppression of cancer stemness, and it could suppress tumor growth and inhibit the metastasis of tumor cells to livers and lungs with therapeutic safety in BALB/C mice [437].

## 7. The Important Health Benefits of Lignan (Sesamin)

Lignans are naturally occurring compounds produced and accumulated in different edible and medicinal plants, which can be subdivided bio-synthetically into neolignan and lignans [438,439]. Lignans, as the notable subgroup of phenylpropanoids, are involved in the plant defense responses to numerous biotic and abiotic stresses [440]. Lignans, with different biological activities, such as antitumor, antibacterial, antioxidant, and antiviral activities, are generally distributed in nature and mostly exist in the xylem of plants [441,442]. The level of lignans varies between plant parts of all species [443].

Sesamin, a major lignan derived from sesame seeds, has several benefits and medicinal characteristics [444]. It exerts various pharmacological impacts, such as prevention of hyperlipidemia, hypertension, and carcinogenesis, as well as anticancer and chemopreventive activity in vitro and in vivo [445,446], and antioxidant and anti-inflammatory characteristics [447,448]. Plants reported to contain sesamin are *Paulownia tomentosa* Staud., *Phyllarthron comorense*, *Justicia simplex*, *Hyptis tomentosa*, *Anacyclus pyrethrum*, *Artemisia absinthium*, *Artemisia gorgonum*, *Chrysanthemum cinerariaefolium*, *C. frutescens*, *C. indicum*, *Diotis maritima*, *Eupatorium ageratina*, *E. ritonia*, *E. fleischmannia*, *Otanthus maritimus*, *Aptosimum spinescens*, *Gmelina arborea* Roxb., *Acanthopanax senticosus*, *A. sessiliflorum*, *Eleutherococcus divaricatus*, *Asarum sieboldii*, *Aristolochia cymbifera*, *Alnus glutinosa*, *Salicomia europaea*, *Austrocedrus chilensis*, *Evodia micrococca*, *Fagara xanthoxyloides*, *Fagara tessmannii*, *Fagara heitzii*, *Micromelum minutum*, *Melicope glabra*, *Spiranthera odoratissima*, *Flindersia pubescens*, *Zanthoxylum naranjillo*, *Zanthoxylum tingoassuiba*, *Zanthoxylum piperitum*, *Zanthoxylum nitidum*, *Zanthoxylum flavum*, *Zanthoxylum alatum* Roxb., *Zanthoxylum bungeanum*, *Ginkgo biloba*, *Machilus glaucescens*, *Ocotea usambarensis*, *Aiouea trinervis* Meisn., *Talauma hodgsonii*, *Magnolia* spp., *Picea abies*, *Macropiper excelsum*, *Piper sarmentosum*, *Sesamum indicum*, *S. radiatum*, *S. mulayanum*, *S. malabaricum*, *S. alatum*, *S. angustifolium*, *S. angolense*, *S. calycinum*, *Anemopsis californica*, *Quercus frainetto* Ten., *Vernicia fordii*, *Jatropha curcas*, *Larrea tridentata*, *Morinda citrifolia*, *Glossostemon bruguieri*, *Ligustrum japonicum*, and *Triclisia sacleuxii* [449]. Sesamin could boost the proliferation and adhesion of intestinal probiotics, leading to modulating gut microbiota, which provided the basis for sesamin as a food-borne functional parameter for improving intestinal health [450]. Sesamin suppressed breast cancer proliferation, and it downregulated programmed death ligand 1 (PD-L1) expression, which is mediated by NF-KB and AKT [451]. Sesamin increased osteoblast differentiation by the increase of type I collagen (COL1A1) and alkaline phosphatase (ALP) gene expression as well as ALP activity [452]. Sesamin ameliorated lead-induced neuroinflammation in rats, and decreased accumulation of lead in blood and neuronal tissues of rats [453]. It ameliorated polymorphonuclear neutrophils infiltration and exudate volume [454]. Majdalawieh et al. [455] reported that sesamin can potentially be utilized as an effectual adjuvant therapeutic agent in ameliorating tumor development and progression, and it could be utilized in the prevention and treatment of different types of cancer. It has been reported that sesamin promoted diabetes-induced neuroinflammation in rats, exhibited neurotrophic supportive action in diabetic rats, and prevented neuronal loss in diabetic rats [456]. Sesamin has a chondroprotective effect through inhibition of proteoglycans (PGs) degradation induced by IL-1beta and inhibition of collagen degradation [457].

## 8. The Health Benefits of Condensed Tannins or Proanthocyanidins (Procyanidin B1)

Proanthocyanidins, also known as condensed tannins [458,459], belong to the oldest of plant secondary metabolites, and these constituents are widespread in woody plants, but are also discovered in certain forages, as well as fruits, seeds, nuts, and bark [460,461]. Yu et al. [462] reported that proanthocyanidins were prevalent in lotus seed coats. They can be categorized into three groups according to their component units and the linkages between them: procyanidins, prodelphinidins, and propelargonidins [463]. The biological activity of plant proanthocyanidins is associated with their chemical concentration and structure [464]. Proanthocyanidins from *Pinus thunbergii* mainly included catechin/epicatechin, and they showed significant antioxidant capacity [465]. Proanthocyanidins in tea, black currant, grapes, bilberry, pine bark, cranberry, and peanut skin may lead to a decrease in the oxidative stress (ROS), induce lower iNOS and COX-2 over-expression, then lower inflammation, and, lastly, show activities against diabetes, asthma, neuropathologies, cardiovascular ailments, obesity, and cancer [466]. The precursors of proanthocyanidins are produced by the phenyl propanoid pathway in the cytosol and are converted to the vacuole, where they polymerize to create proanthocyanidins [467]. They have various bioactivities, such as anticancer, antibacterial, and antioxidant [468]. Proanthocyanidins stimulate antioxidant capacity and increase resistance against oxidative stress-induced senescence in fruits after harvest [469].

Procyanidins are associated with the class of natural products known as proanthocyanidins or condensed polyphenols [470]. They have been reported to reveal broad advantages to human health and are applied in the prevention of cancers, diabetes, cardiovascular diseases, etc. [471]. They are structurally diverse constituents and can be divided into monomeric, oligomeric, or polymeric variants associated with degree of polymerization, which plays a role in manifesting various impacts that are associated with human health [471]. The anti-digestion and antioxidant impacts of grape seed procyanidins have been proven [472]. Procyanidin B1 is also a promising liver cancer antitumor drug [473] (Na et al., 2020). Procyanidins increase the glycometabolism and decrease the secretion of inflammatory factors of postpartum mice with gestational diabetes mellitus (GDM) [474].

## 9. The Health Benefits of Curcuminoids (Curcumin, Demethoxycurcumin, Bisdemethoxycurcumin)

### 9.1. Curcuminoids

Curcuminoids are a group of polyphenol coloring constituents that exist in the plant species *Curcuma*, such as *Curcuma longa*, *C. Wenyujin*, *C. zedoaria*, etc. [475,476]. They are synthesized in turmeric from cinnamic acid precursors obtained via the phenylpropanoid biosynthetic pathway, and there are three different precursors, namely curcuminoids biosynthesis-cinnamic acid, ferulic acid, and coumaric acid [477]. Ramirez-Ahumada et al. [478] reported that curcuminoid synthase activity in turmeric crude protein extracts converts feruloyl-CoA into curcumin. Curcumins are the commercially available component in curcuminoids, as the principle constituents, and the other two, demethoxycurcumin and bisdemethoxycurcumin, as minor components [479,480]. Curcumin and demethoxycurcumin are distinctive because of the phenylmethoxy group [481]. Curcuminoids share important pharmacological characteristics possessed by turmeric, a distinguished curry spice, considered as an important factor in Alzheimer’s disease [482]. It has been reported that curcuminoids of turmeric can be considered as a modern medicine for the treatment of knee osteoarthritis [483] as well as a potential anticancer agent [484]. Zhou et al. [485] also reported that turmeric rhizomes exhibit versatile biological activities such as a significant anticancer property. Three curcuminoids, namely curcumin, demethoxycurcumin, and bisdemethoxycurcumin, in turmeric were found and were shown to contain significant synergistic anticancer activities [486]. Curcuminoids rescued neurotoxin-induced inflammatory gene expression and rescued neurotoxin-induced apoptotic gene expression, and individual curcuminoids showed significant function useful for Alzheimer’s disease [482].

### 9.2. Curcumin

Curcumin (bis-α,β-unsaturated β-diketone), also known as diferuloylmethane, is a hydrophobic polyphenol obtained from the rhizome of the perennial herb genus *Curcuma,* which belongs to the ginger family (Zingiberaceae) and consists of species such as *Curcuma longa*, *Curcuma amada*, *Curcuma aromatic*, *Curcuma zedoaria*, and *Curcuma raktakanta* [487,488]. Curcumins contain different medicinal values such as antioxidant, anti-pulmonary fibrosis, anti-inflammation, antiviral, and chronic obstructive pulmonary disease impacts, and attractively docked with multi-target molecular proteins related to diabetes [489,490,491,492,493,494]. Curcumin is insoluble in water and easily efficient in organic solvents [495]; the active functional groups of curcumin can be oxidized by electron transfer and hydrogen abstraction [496], and curcumin is more durable in acidic to neutral conditions than in alkaline circumstances [495,496,497]. Curcumin, as an enzyme inhibitor, has proper structural characteristics including a flexible backbone, hydrophobic nature, and different available hydrogen bond (H-bond) donors and acceptors [498]. Curcumin is stable to heat but is light-sensitive and produces singlet oxygen and other reactive oxygen species (ROS) when exposed to the sun, which is also a photodynamic and photobiological property of curcumin [499]. Curcumin decreases inflammation by inhibiting lipopolysaccharide-induced nuclear factor-KB (NF-KB) p65 translocation and mitogen-activated protein kinase activation in dendritic cells [500]. Curcumin decreases morphine dependence in rats through an inhibitory influence on neuroinflammation and a decline in the expression of μ-opioid receptors in the prefrontal cortex [501]. Curcumin influences synaptic plasticity genes (Arc and Fmr1) to decrease amnesia [502]. Xie et al. [503] reported that curcumin together with photodynamic therapy have been confirmed as effective in many kinds of cancer cells in vitro and animal models. It has been extensively applied in cancer treatment because of its ability to trigger cell death and suppress metastasis [504]. Mahjoob and Stochaj [505] reported that curcumin improves aging-related cellular and organ dysfunctions. Curcumin can be a promising antifatigue substitute for improving exercise performance [506]. Its derivatives have anti-inflammatory actions for drug repurposing in traumatic brain injury (TBI), but their molecular targets are not clear [507].

### 9.3. Demethoxycurcumin

Demethoxycurcumin is one of the principle active compounds of curcuminoids discovered in turmeric powder, which is used as a spice in Asian cooking and traditional medicine [508]. Recent studies reveal that demethoxycurcumin has various biological activities including antioxidant, anti-inflammation, and anticancer activities [509,510,511]. Lin et al. [512] reported that demethoxycurcumin is the most active constituent against various kinds of breast cancer cell lines and induces apoptosis and autophagy. Demethoxycurcumin, a natural derivative of curcumin, revealed stronger inhibitory activity on nitric oxide and tumor necrosis factor-α production in comparison with curcumin in lipopolysaccharide-activated rat primary microglia [513]. Demethoxycurcumin remitted the inflammation of nucleus pulposus cells without overt cytotoxic impacts [514].

### 9.4. Bisdemethoxycurcumin

Bisdemethoxycurcumin is a demethoxy derivative of curcumin and is much more stable than curcumin in physiological media [514,515,516]. It can scavenge free radicals and control cellular redox balance because of its antioxidant property [517,518], and it has potential anti-allergic effects [519]. Mahattanadul et al. [520] reported that bisdemethoxycurcumin’s antiulcer impacts might be because of its characteristics of decreasing gastric acid secretion and increasing the mucosal defensive mechanism via suppression of inducible nitric oxide synthase (iNOS)-mediated inflammation. Bisdemethoxycurcumin inhibits human pancreatic α-amylase (HPA) [521].

## 10. Conclusions

Phenolic compounds are one of the most important types of compounds with an important role in growth and reproduction, providing protection against pathogens and predators, and they could be the main determinant of antioxidant potential of foods. Phenolics are a heterogeneous collection of compounds generated as secondary metabolites in plants. Phenolic compounds are aromatic or aliphatic compounds with at least one aromatic ring to which one or more OH groups are connected. They are subdivided into different groups depending on the number of phenolic rings that they possess and the structural elements joined to them. They are naturally occurring compounds present in several foods such as cereals, fruits, vegetables, and beverages. Polyphenols can also be found in dried legumes and chocolate. The distribution of phenolic compounds in plant tissues and cells change considerably according to the type of chemical compound. They also contribute towards the color and sensory characteristics of fruits and vegetables. Different classes of phenolic compounds in plants are simple phenolics, benzoquinones, hydroxybenzoic acids, acetophenones, phenylacetic acids, hydroxycinnamic acids, phenylpropanoids, naphthoquinones, xanthones, stilbenes, anthraquinones, flavonoids, isoflavonoids, lignans, neolignans, biflavonoids, lignins, and condensed tannins. Hydroxybenzoic acids are gallic acid and Protocatechuic acid. Hydroxycinnamic acids are *p*-coumaric acid, caffeic acid, ferulic acid, sinapic acid, and other components such as coumarins (umbelliferone, esculetin, scopoletin, resveratrol, piceatannol, pterostilbene), curcuminoids (curcumin, demethoxycurcumin, bisdemethoxycurcumin), condensed tannins or proanthocyanidins (procyanidin B1), and lignan (sesamin). From a human physiological viewpoint, phenolic compounds are important in defense responses such as antioxidant, anti-aging, antiproliferative, and anti-inflammatory. High phenolic activity in many species could prove to be beneficial towards human health if included as part of food designs for a healthy diet.

Flavonoids are the largest group of natural phenolic compounds, and, based on the differences in the pyran ring, flavonoids can be divided into flavones, isoflavones, flavanonols, flavonols, flavanones, flavan-3-ols, and anthocyanidins. They can be subdivided into different subgroups on the basis of the carbon of the C ring on which the B ring is attached and the degree of unsaturation and oxidation of the C ring. Flavonoids in which the B ring is linked in position 3 of the C ring are called isoflavones. Those in which the B ring is linked in position 4 are called neoflavonoids, while those in which the B ring is linked in position 2 can be further subdivided into several subgroups on the basis of the structural characteristics of the C ring. The most prominent health benefits of phenolic compounds are antioxidant activity, anti-inflammatory properties, antifungal activity, antimicrobial activity, antibacterial properties, anti-coronavirus activities, neuroprotective potential, appropriate for skin health, suitable for wound healing, and anticancer activities. Flavonoids, a group of natural substances with variable phenolic structure, are found in vegetables, fruits, grains, bark, stems, roots, flowers, wine, and tea. Flavonoids are considered as an important constituent in different pharmaceutical, medicinal, nutraceutical, and cosmetic applications. They belong to a class of low-molecular-weight phenolic compounds that are extensively distributed in the plant kingdom. Future research is needed to determine the pharmaceutical benefits of phenolic and flavonoid compounds of medicinal plants, especially traditional Chinese medicinal plants, and to gain a better understanding of these chemical compounds in medicinal plants and herbs. It is also important to increase analytic techniques to allow the collection of more data on excretion and absorption.

## Figures and Tables

**Table 1:** Classes of phenolic compounds in plants [53].

Class	Structure
Simple phenolics, benzoquinones	C_6_
Hydroxybenzoic acids	C_6_-C_1_
Acetophenones, phenylacetic acids	C_6_-C_2_
Hydroxycinnamic acids, phenylpropanoids (coumarins, isocoumarins, chromones, chromenes)	C_6_-C_3_
Napthoquinones	C_6_-C_4_
Xanthones	C_6_-C_1_-C_6_
Stilbenes, anthraquinones	C_6_-C_2_-C_6_
Flavonoids, isoflavonoids	C_6_-C_3_-C_6_
Lignans, neolignans	(C_6_-C_3_)2
Biflavonoids	(C_6_-C_3_-C_6_)2
Lignins	(C_6_-C_3_)_n_
Condensed tannins (proanthocyanidins or flavolans)	(C_6_-C_3_-C_6_)_n_

**Table 2:** Examples of hydroxybenzoic and hydroxycinnamic acids.

Phenolic Acids	Examples	Molecular Formula
**Hydroxybenzoic acids**	Gallic acid Protocatechuic acid	C_7_H_6_O_5_ C_7_H_6_O_4_
**Hydroxycinnamic acids**	*p*-coumaric acid Caffeic acid Ferulic acid Sinapic acid	C_9_H_8_O_3_ C_9_H_8_O_4_ C_10_H_10_O_4_ C_11_H_12_O_5_
**Other components**		
**Coumarins**	Umbelliferone Esculetin Scopoletin	C_9_H_6_O_3_ C_9_H_6_O_4_ C_10_H_8_O_4_
**Stilbenes**	Resveratrol Piceatannol Pterostilbene	C_14_H_12_O_3_ C_14_H_12_O_4_ C_16_H_16_O_3_
**Curcuminoids**	Curcumin Demethoxycurcumin Bisdemethoxycurcumin	C_21_H_20_O_6_ C_20_H_18_O_5_ C_19_H_16_O_4_
**Condensed tannins or proanthocyanidins**	Procyanidin B1	C_30_H_26_O_12_
**Lignan**	Sesamin	C_20_H_18_O_6_

**Table 3:** Generic structure of major classes of flavonoids.

Flavonoids		Molecular Formula
**Flavones**	Apigenin Luteolin Chrysin	C_15_H_10_O_5_ C_15_H_10_O_6_ C_15_H_10_O_4_
**Flavonols**	Kaempferol Quercetin Isorhamnetin	C_15_H_10_O_6_ C_15_H_10_O_7_ C_16_H_12_O_7_
**Flavanones**	Naringenin Eriodictyol Hesperetin	C_15_H_12_O_5_ C_15_H_12_O_6_ C_16_H_14_O_6_
**Flavanols**		C_15_H_14_O_2_
**Anthocyanidin**		C_15_H_11_O^+^
**Flavanonols**	Taxifolin Aromadendrin	C_15_H_12_O_7_ C_15_H_12_O_6_
**Flavan-3-ols**	Gallocatechin Catechin	C_15_H_14_O_7_ C_15_H_14_O_6_
**Isoflavones**	Genistein Daidzein Formononetin	C_15_H_10_O_5_ C_15_H_10_O_4_ C_16_H_12_O_4_

**Table 4:** Important points about phenolic acids and their derivatives.

The Derivatives of Phenolic Acids	Key Points	References
**Flavonoids**	The largest group of natural phenolic compounds.	[54,114]
	Their structure is based on a 15-carbon phenyl benzopyran skeleton (C6-C3-C6, i.e., A-C-B rings).	[54,114]
	Based on differences in the pyran ring, flavonoids can be categorized into flavones, isoflavones, flavanonols, flavonols, flavanones, flavan-3-ols, and anthocyanidins.	[54,114]
	The majority occur as glycosides, except for flavan-3-ols, which are rarely glycosylated.	[54,114]
	Different patterns of hydroxylation and methylation of the A and B rings consequently result in a variety of compounds for each flavonoid category.	[54,114]
	Flavones have a double bond between C-2 and C-3, a keto function in C-4, and the B ring is attached at C-2.	[54,114]
	The most common flavonoes in medicinal and aromatic plants are luteolin, apigenin, and glycosides.	[54,114]
	In isoflavones, the B ring is attached at C-3 and the main components are daidzein, genistein, and glycitein.	[54,114]
	Flavonols are flavones bearing a hydroxyl group at C-3, such as kaempferol, quercetin, and myricetin.	[54,114]
	In flavanones, the C-ring has no double bond between C2 and C3, such as in naringenin, eriodictyol, and hesperetin.	[54,114]
	Flavanonols, also called dihydroflavonols, have the same saturated C-ring as flavanones but are hydroxylated at C-3.	[54,114]
	Flavan-3-ols, also referred to as flavanols, also contain a saturated C-ring, but lack the keto group at C-4, and are hydroxylated at C-3, such as catechin and gallocatechin, or as oligomers and polymers.	[54]
	In anthocyanidins, the C-ring lacks the keto group at C-4, is hydroxylated at C-3, and, uniquely, has two double bonds forming the flavylium cation, such as in cyanidin, petunidin, malvidin, pelargonidin, peonidin, and delphinidin.	[54]
**Stilbenes**	They are based on 1,2-diphenylethylene, which has a C6-C2-C6 skeleton.	[115]
	They can be found as aglycones, monomers, oligomers, or glycosylated derivatives.	[116]
**Tannins**	Tannins are high molecular weight polyphenolic compounds.	[117,118]
	They can be synthesized as a defensive mechanism in response to pathogen attack and abiotic stresses such as UV radiation.	[117,118]
	Based on their structures, tannins in plants can be classified into mainly hydrolysable tannins and condensed tannins, also known as proanthocyanidins.	[117,118]
	Hydrolysable tannins are built based on gallic acid and are divided into the gallotannins and ellagitannins.	[117,118]
**Quinones**	They contain a di-one or di-ketone group.	[119]
	They are distinguished into benzoquinones and naphthoquinones and are based on their derivative molecules.	[119]
	They may occur as monomers, dimers, trimers, glycosides, or in reduced forms.	[119]
**Coumarins**	They may occur in a free or glycosylated state.	[120]
	They are divided into six categories, namely simple coumarins, furanocoumarins, dihydrofuranocoumarins, pyranocoumarins, phenylcoumarins, and bicoumarins.	[120]
**Curcuminoids**	They widely occur in *Curcuma* spp., especially in the rhizomes of *Curcuma longa* (turmeric).	[121,122]
	There are three major curcuminoids, namely curcumin, demethoxycurcumin, and bis-demethoxycurcumin.	[121,122]
	The structure of curcumin consists of a keto-enol tautomeric unsaturated chain linking two aromatic rings bearing a hydroxyl and methoxy group.	[121,122]
**Lignins**	Lignans consist of two phenylpropane units joined together by a *β*-*β*′ bond.	[123]
	They are divided into eight categories, namely dibenzylbutyrolactols, dibenzocyclooctadienes, dibenzylbutanes, dibenzylbutyrolactones, arylnaphthalene, aryl-tetralins, furans, and furofurans.	[123]

**Table 5:** Biological activities of phenolic compounds.

Health Benefits	Key Points	References
**Antioxidant activity**	* The stem of *Dendrophthoe falcata* (Loranthaceae) plant had a high content of phenolic and flavonoid compounds and very high antioxidant activities.	[182]
	* The phenolic compounds of *Buchenavia tetraphylla*, *Buchenavia tomentosa*, and *Lippia sidoides* provided the main contributions to the antioxidant potential.	[183]
	* The total phenolic, flavonoid, and antioxidant capacity of all blueberry cultivars increased nonlinearly with ripening.	[184]
	* Cynaroside, rosmarinic acid, cosmosiin, luteolin, apigenin, and acacetin were the main components in ethyl acetate extracts of *Salvia absconditiflora*, *Salvia sclarea*, and *Salvia palaestina* with antioxidant activity.	[185]
	* Phenolic compounds from *Pistacia lentiscus* L. black fruits exhibited potent antioxidant properties.	[186]
	* Lycium berries of different species contained a total of 186 phenolic compounds, which exhibited potent antioxidant activities.	[187,188]
	* Stachys species contained important bioactive phenolics and had promising antioxidant impacts.	[189]
	* *Acacia nilotica* pods and bark had potent total phenolic content, antioxidant activity, and tyrosinase inhibitory properties.	[190]
	* *Bersama abyssinica* (Meliathacea) was rich in phenolic compounds, flavonoids and coumarin, and 7,8-Dimethoxycoumarin with high antioxidant activity.	[191]
	* Epicatechin was the main monomeric polyphenol in the profile of longan phenolics.	[192]
	* Epicatechin, quercetin 3-*O*-rhamnoside, and kaempferol were responsible for the high antioxidant activity of *Litsea glaucescens*.	[193]
	* The water extract of *Amsonia orientalis* leaves exhibited promising antioxidant activity when used at low concentration.	[194]
	* The ethanolic extract of *Amsonia orientalis* leaves had the highest phenolic substance content and 2,2-diphenyl-1-picrylhydrazyl (DPPH) scavenging activity.	[194]
**Anti-inflammatory activity**	* A variety of phenolic compounds and stilbene derivatives in different parts of germinated peanut suggested that the peanut sprout exerted high anti-inflammatory effects and may be related to the polyphenolic content and antioxidant properties.	[195]
	* Fermented olive cream and *Lactiplantibacillus* (Lpb.) plantarum IMC513 reduced proinflammatory cytokine levels.	[196]
	* *Allium scorodoprasum* L. subsp. *rotundum* extract showed high anti-inflammatory inhibitory effects against xanthine oxidase activity.	[197]
	* *Helleborus purpurascens* demonstrated the strongest anti-inflammatory potential, especially because of fatty acids.	[198]
	* *Thalictrum minus* possessed combined anti-inflammatory and antioxidant effects.	[198]
	* The leaf of *Aurea helianthus* demonstrated strong anti-inflammatory activity that reduced NO production.	[199]
**Antifungal activity**	* *Hypericum empetrifolium* aerial parts extract (HEA) exhibited antifungal activity against *Candida tropicalis* with 19.53 μg/mL.	[200]
	* *Allium sativum* extract revealed strong antifungal activity effects against *Curvularia* spp., *Trichophyton* spp., and *Geotrichum* spp.	[201]
	* *Rosa micrantha* flowers extract revealed fungicide effects in *Candida glabrata*.	[202]
	* Phenolic compounds of *Ulmus davidiana* var. *japonica* showed antifungal activity against *Cryptococcus neoformans* and *Candida albicans*.	[203]
	* *Zataria multiflora* essential oils could act as natural fungicides; carvacrol and thymol chemotypes of *Zataria multiflora* inhibited five important fungal plant pathogens.	[204]
	* *Aconitum heterophyllum* and *Polygonum bistorta* exhibited significant antimicrobial and antioxidant activity.	[205]
**Antimicrobial activity**	* The antimicrobial activities of mint and thyme were due to a wide range of diverse phenolics such as menthone, menthyl acetate, menthol, terpenes, and thyme.	[206]
	* Phenolic compounds of *Codonopsis lanceolata* plants exhibited notable antimicrobial activity.	[207]
	* Phenolic compounds of cashew (*Anacardium occidentale* L.) compounds identified included mainly flavanols, which showed high antimicrobial activity.	[208]
	* *Ixora coccinea* Linn. root contained bioactive phenolic compounds including pyrocatechol, catechin, and chlorogenic acid with potent antimicrobial effects.	[209]
**Antibacterial activity**	* The ethyl acetate fraction of *Scirpus holoschoenus* showed the highest antioxidant activity and antibacterial effect for *Staphylococcus aureus* and *Bacillus subtilis*.	[210]
	* *Rhanterium adpressum* showed antibacterial activity.	[211]
	* The lignum of *Rhus verniciflua* contained high content of phenolic compounds with less urushiols, which suggests efficient antibacterial activity with less toxicity.	[212]
	* Phenolic compounds of *Scrophularia ningpoensis* Hemsl. showed antibacterial activity.	[213]
	* Flavonoids, saponin, alkaloids, tannins, steroids, and terpenoids of *Solanum incanum* L. and *Harrisonia abyssinica* Oliv. exhibited antibacterial activity.	[214]
	* The phenolic extracts from *Cerbera manghas*, *Commelina diffusa*, *Peperomia pellucida*, *Kleinhovia hospita*, *Mikania micrantha*, *Homalanthus nutans*, *Psychotria insularum*, *Phymatosorus scolopendria*, *Piper graeffei*, and *Schizostachyum glaucifolium* exhibited antibacterial activities.	[215]
**Anti-Coronavirus Properties**	* Curcumin has been suggested as a potential treatment choice for patients with COVID-19 because it inhibits ACE2 and suppresses the entry of SARS-CoV-2 into the cells.	[216]
	* Theaflavin, the compound responsible for the orange/black color of black tea, is a potent inhibitor of the RNA polymerase of SARS-CoV-2.	[217]
	* Catechin gallate and gallocatechin gallate also showed high inhibitory activity against SARS-CoV-2 N protein in a concentration-dependent manner and affected virus replication.	[218]
	* Myricetin could be further tested and developed as a potential SARS-CoV-2 antiviral.	[219]
	* The phenolic compounds *Kadsurenin* L. and Methysticin of *Piper nigrum* are candidate ligands for inhibiting COVID-19.	[220]
	* Plant-derived phenolic compounds of *Isatis indigotica* root were frequently used for the prevention of SARS during the SARS outbreaks in east Asia.	[221]
	* Among phenolic acid constituents, chlorogenic acid, caffeic acid, and gallic acid of *Sambucus Formosana Nakai* reduced cytopathicity and virus yield in HCoV-NL63-infected cells.	[222]
	* Some phenolic compounds such as diethylstilbestrol, enterodiol, enterolactone, flavokawain A, flavokawain B, and flavokawain C showed excellent to good inhibitory activities against collagenase, elastase enzymes, and SARS-CoV-2.	[223]
	* The phenolic compounds of blackcurrant (*Ribes nigrum* L.) had antiviral activity in Coxsackievirus A9 and human coronavirus HCoV-OC-43.	[224]
**Neuroprotective potential**	* Hydroxytyrosol obtained from olive exhibited neuroprotective impacts on multiple chronic neurodegenerative diseases including Alzheimer’s, Parkinson’s, and multiple sclerosis.	[225]
	* The protective impacts of oil palm phenolics against neurodegenerative diseases have been recently identified.	[226]
	* Phenolic compounds of *Hypericum wightianum*, namely Hyperwightin E and petiolin G, revealed noticeable neuroprotection at 10 μM.	[227]
	* *Inula viscosa* (L.) Greuter has high total phenolics and flavonoids and demonstrated neuroprotective properties.	[228]
	* *Maclura tinctoria* leaf aqueous extract contained high phenolic components, and it has been found that neuroprotective effects of it could be associated with the presence of the phenolic compounds identified.	[229]
**Skin health**	* Phenolic compounds from *Lippia microphylla* and *Dimorphandra gardneriana* presented a high sun protector factor because of the presence of sakuranetin flavonoids and quercetin glycosides.	[230]
	* Among Moroccan medicinal plants, *Allium cepa* L., *Chamaeleon gummifer* (L.) Cass, and *Salvia rosmarinus* Schleid. Mill. leaves were the most commonly used for different types of skin diseases.	[231]
	* *Panax ginseng* C.A. Meyer and *Nardostachys chinensis* Bat. have been confirmed for the treatment of skin pigmentary disorders.	[232]
	* The protective effects on skin cells associated with blueberry phenolic compounds that included inhibition of proliferation and cell cycle arrest in malignant cells decreased oxidized macromolecules, down-regulated inflammatory cytokine genes, and mitigated oxidative stress.	[233]
**Wound healing**	* Gel containing Ipomoea pes-caprae (Ipc) phenolic-rich leaf extract accelerated the wound-healing process.	[234]
	* *Loranthus acaciae* exhibited high phenolic contents and wound healing activities.	[235]
	* *Haworthia limifolia* showed excellent wound-healing properties because of high phenolic contents.	[236]
	* *Lawsonia inermis* and *Azadirachta indica* are well known for wound healing.	[237]
	* Aloe vera (*Aloe barbadensis*) is one of the oldest medicinal plants with wound healing activity for a variety of skin disorders including burns as well as infections and diabetic dermal wounds.	[238]
	* *Amphimas pterocarpoides* leaves and stem bark have high phenolic and flavonoid contents, and it has been proven that leaf and stem bark ointments increased the rate of wound healing in rats.	[239]
**Anticancer activity**	* *Sedum dendroideum* showed anti-proliferative activity in breast cancer cells.	[240]
	* *Hypericum perforatum* extract exhibited a remarkable total phenol content, which showed high anticancer activity.	[241]
	* *Ficus palmata* Forssk. exhibited high total phenolic and flavonoids contents, which showed its high anticancer activity.	[242]
	* *Euphorbia thymifolia* and *Euphorbia hirta* showed anticancer activity against ascites carcinoma in mice models.	[243]
	* *Vitis vinifera* L. contained high phenolic components, which can be considered as a promising potential for an anticancer drug.	[244]
	* Phenolic compounds and alkaloid compounds of *Dysphania ambrosioides* might have significantly contributed to anticancer activity.	[245]
	* *Sisymbrium officinale* had considerable phenolic and flavonoids contents, which was why it showed anticancer activity.	[246]

**Table 6:** Coumarin-based compounds obtained from the medicinal plants used by various ancient medical systems [340].

Compounds	Molecular Formula	Pharmaceutical Activity
6-hydroxy-4-methoxy-5-methylcoumarin	C_11_H_10_O_4_	Microtubule stabilizing agent
(+)-Calanolide	C_22_H_26_O_5_	Anti-HIV agent
Inophyllum	C_25_H_24_O_5_	Anti-HIV agent
Theraphin	C_22_H_28_O_6_	Anticancer agent Antimalarial agent

## Data Availability

Not applicable.
